# Efficacy of Thiamine in the Treatment of Postcardiac Arrest Patients: A Randomized Controlled Study

**DOI:** 10.1155/2020/2981079

**Published:** 2020-06-08

**Authors:** Suntornwit Pradita-ukrit, Veerapong Vattanavanit

**Affiliations:** Critical Care Medicine Unit, Division of Internal Medicine, Faculty of Medicine, Prince of Songkla University, Hat Yai, Songkhla 90110, Thailand

## Abstract

**Background:**

Thiamine administration has been shown to improve survival in a postcardiac arrest animal study. We aimed to evaluate the efficacy of thiamine in comatose out-of-hospital cardiac arrest (OHCA) patients following return of spontaneous circulation.

**Methods:**

A randomized, double-blinded, placebo-controlled study was conducted. Thirty-seven OHCA patients were randomly assigned to receive either thiamine 100 mg every 8 hours or a placebo. The primary outcome was 28-day all-cause mortality.

**Results:**

Over the course of 2 years, 37 patients were randomized to either receive thiamine (*n* = 20) or a placebo (*n* = 17). The primary outcome was not different between the groups: 10/20 (50%) in the thiamine group vs. 8/17 (47.1%) in the placebo group (*P*=0.93 by the log-rank test). There were no significant differences in secondary outcomes between the groups (good neurological outcome, lactate level, and S100B level).

**Conclusions:**

In this study, there were no significant differences in survival outcome. Further studies with a larger population are necessary to confirm these results.

## 1. Introduction

Out-of-hospital cardiac arrest (OHCA) is a leading cause of death worldwide. An estimated 350,000 American adults suffer from cardiac arrest each year [1]. Although advanced resuscitation systems and intensive care management have been developed, the proportion of survival to hospital discharge is reported to be only 10.4% [1]. Neurological injury is the leading cause of death in patients who are initially successfully resuscitated [2, 3].

Survival of OHCA patients depends on the chain of survival, which includes immediate recognition of cardiac arrest and activation of the emergency medical system, immediate high-quality cardiopulmonary resuscitation (CPR), rapid defibrillation, basic and advanced medical services, advanced life support, and postcardiac arrest care [4]. After return of spontaneous circulation (ROSC), the treatment of the specific cause of cardiac arrest, coronary intervention if indicated, and targeted temperature management in comatose patients are recommended, with the intention of reducing brain injury and improving survival [5]. The combination of vasopressin, epinephrine, and steroids has been proven to benefit the survival and neurological outcomes, but only in in-hospital cardiac arrest patients [6]. Hypoxic-ischemic encephalopathy is the leading cause of death among postcardiac arrest patients. Hypoxic-ischemic reperfusion injury is proposed to be a key mechanism of brain injury [7]. A study showed mitochondrial dysfunction, resulting in impaired aerobic metabolism and aggravation of oxidative stress after cardiac arrest [8]. The enzyme pyruvate dehydrogenase (PDH) plays a major role in linking glycolysis with the tricarboxylic acid (TCA) cycle. The activity of PDH and other key enzymes in the TCA cycle is modulated by the essential coenzyme thiamine pyrophosphate. Thiamine administration can restore PDH activity. Ikeda and colleagues [9] showed the benefit of thiamine administration in a mouse model of cardiac arrest.

To the best of our knowledge, this study is the first to evaluate the effect of thiamine administration on the outcomes OHCA patients.

## 2. Methods

### 2.1. Study Design

We conducted an investigator-initiated, single-center, double-blinded, randomized, parallel group, placebo-controlled pilot study between December 2016 and December 2018 in the medical intensive care unit (ICU) of Songklanagarind Hospital, Hat Yai, Thailand. The 800-bed hospital is the largest university-based tertiary care center in Southern Thailand. The Emergency Medical Services (EMS) in Thailand is a two-tiered ambulance system. The life support is provided by either hospital-based ambulances or nonpublic health sector organizations [10]. However, some patients were transported to the hospital in private vehicles without CPR or without activation of the EMS. No mechanical compression device was used at the scenes. The quality of CPR was not routinely monitored in every case. The 24 h call dispatch center was manned by trained personnel provided by the government.

**Figure 1:**
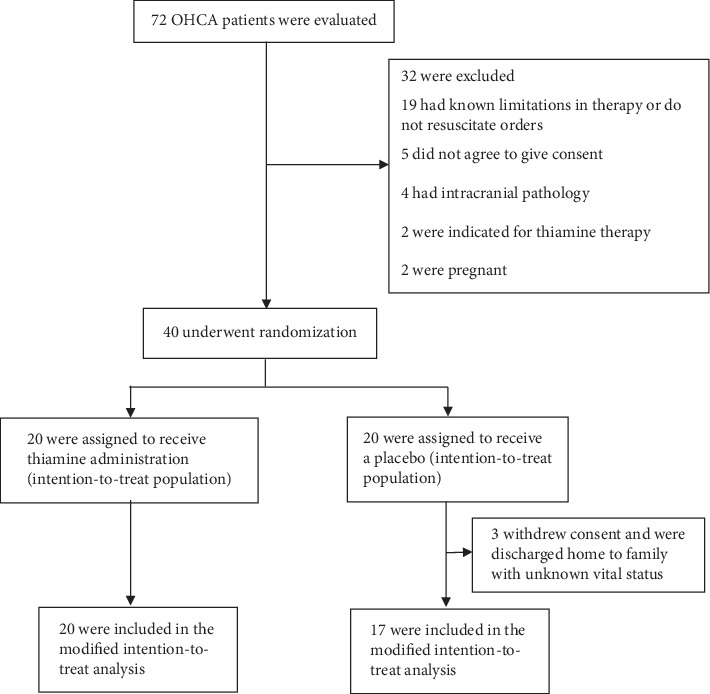
Flow diagram describing the screening, recruitment, and randomization of patients. OHCA, out-of-hospital cardiac arrest.

The study protocol was approved by the Institutional Review Board (REC 59-289-14-1) and registered with the Thai Clinical Trial Registry (TCTR20161226001). An independent data safety monitoring board monitored the study for complications, with predetermined discontinuation criteria (see in Supplementary Appendix (available (here))). The study complied with all of the principles set forth in the Declaration of Helsinki (1964) and its subsequent provisions. Informed consent to participate was obtained from next of kin or legal guardian of the patients prior to inclusion in the study at the medical ICU. All screening and enrollment of participants was performed by the investigators (Figure 1). The outcome evaluation, data management, and analysis were conducted by the investigators and statisticians, both of whom were blinded to the patient enrollment and treatment processes.

### 2.2. Patients

We prospectively screened patients aged 18 years or older who were successfully resuscitated from OHCA. Patients who were comatose (Glasgow Coma Scale (GCS) score of 8 or less) and admitted to the medical ICU were eligible. The exclusion criteria included known limitation in therapy, pregnancy, being comatose from intracranial pathology (ischemic stroke or intracerebral hemorrhage), poor neurological performance status, prior cardiac arrest, and contraindications to thiamine. A full list of inclusion, exclusion, and discontinued criteria is provided in the Supplementary Appendix (available (here)).

### 2.3. Randomization and Study Intervention

The investigators evaluated patients for eligibility, obtained informed consent, and enrolled the participants. After inclusion, patients were randomly assigned without restriction in a 1 : 1 ratio (thiamine and placebo) according to a computer-generated randomization table derived from http://www.randomization.com by a research nurse assistant, who had no role in patent management. The research nurse assistants that were not otherwise involved in the study administrated both the study drug and placebo. The attending physicians, nursing care teams, research investigators, and participants and their family members were blinded to treatment allocation. The study drug (thiamine or placebo) was prepared by a pharmacist who had no other role in the trial. The study drugs were package in nonidentical 100 mL piggy bags labelled with sequential numbers. Thiamine hydrochloride (ANB Laboratories Co., Ltd.) was given daily as an intravenous bolus (100 mg in 100 mL of normal saline) every 8 h for 7 days. The control group received a comparable volume of normal saline on the same schedule. The standard postcardiac arrest care in the medical ICU was provided according to the 2015 guidelines [5]. Performing coronary intervention and/or targeted temperature management were decided by attending cardiologists and intensivists, respectively.

### 2.4. Study Endpoints and Data Collection

The primary endpoint of the study was all-cause 28-day mortality. Secondary outcomes included good neurological outcome (cerebral performance category (CPC) 1–2) at hospital discharge, serum lactate level and clearance at 24, 48, and 72 h, S100B level at 72 h, and ICU length of stay.

Baseline data were collected, including age, gender, comorbidities, location of cardiac arrest, first monitored electrocardiography rhythm by the EMS or at the emergency department, CPR time, Acute Physiology and Chronic Health Evaluation (APACHE) II score, procedures during admission, and complications. We also collected baseline thiamine levels using a high-performance liquid chromatography- (HPLC-) based method from whole blood. For adverse events related to thiamine, we monitored for signs of thrombophlebitis and anaphylaxis.

All patients were followed for up to 28 days or death, whichever occurred first. Data for the primary outcome and the neurological evaluation at 28-day follow-up were obtained from inpatients, in-hospital visits, or telephone contact with patients, relatives, or general practitioners by the investigators. The clinical data of patients was collected from the electronic hospital information system. Good neurological outcome was defined as a CPC of 1 or 2. The CPC scale ranges from 1 to 5, with 1 representing good cerebral performance or minor disability, 2 representing moderate disability, 3 representing severe disability, 4 representing coma or vegetative state, and 5 representing brain death [11].

### 2.5. Statistical Analysis

A sample of 40 patients provided 80% power at a two-sided alpha error of 0.05 to detect a difference of 40% in 28-day mortality, assuming a baseline mortality of 90% [12]. There was no planned interim analysis.

The study was analyzed on a modified intention-to-treat basis, excluding randomized patients with unknown vital status. We used the Wilcoxon rank-sum test for continuous variables and Fisher's exact test, where appropriate, for categorical variables. The primary endpoint was evaluated by Fisher's exact test, and the secondary endpoints were analyzed by Fisher's exact test and Wilcoxon–Mann–Whitney test. For survival analysis, the time to an event of interest was calculated from the date of randomization to the date of the event. Patients who did not experience the event were censored at their hospital discharge. Survival distributions were estimated using the Kaplan–Meier method and compared by the log-rank test.

A *P* value <0.05 was considered to indicate statistical significance for all comparisons. All statistical analyses were performed with STATA version 16 (StataCorp, College Station, TX, USA).

## 3. Results

### 3.1. Patients

During the period from December 2016 through December 2018, a total of 72 patients who had been resuscitated after cardiac arrest were enrolled (Figure 1). After the exclusion of 32 patients, 40 patients were randomized into either the thiamine group or the placebo group. Three patients in the placebo group later withdrew their consent to participate due to their families being willing to take them back home with unknown vital status. Of the remaining patients, 20 in the thiamine group and 17 in the control group were analyzed according to modified intention-to-treat basis.

The baseline characteristics of patients are shown in Tables 1 and S1. The mean (±standard deviation (SD)) age of the patients was 64.3 ± 14.2 years and 70% were men. Cardiac causes accounted for 70% of OHCA cases, and half of patients (19/37, 51%) had a first monitored shockable rhythm. Thirty-six patients (97%) collapsed in front of a witness, but only 19 patients (51%) received bystander CPR. The median time from cardiac arrest to CPR was 5 min, and the median (interquartile range (IQR)) CPR time was 14 (8–24.5) min. There were no differences between the two groups, except that more patients in the thiamine group were diagnosed with STEMI than in the placebo group. Eighteen patients (48%) underwent coronary angiography, and 16 patients (43%) were given targeted temperature management (Tables 1 and S2).

### 3.2. Outcomes

**Figure 2:**
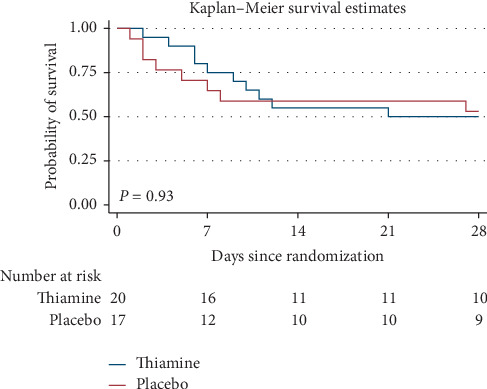
Kaplan–Maier analysis of 28-day survival in out-of-hospital cardiac arrest (OHCA) patients receiving thiamine or placebo.

Data on primary and secondary outcomes are shown in Table 2. By day 28, there was no significant difference in all-cause mortality between the treatment and placebo groups (50% vs. 47.1%; relative risk (RR) 1.06, 95% confidence interval (CI) 0.54–2.07, *P*=0.86). A Kaplan–Meier curve is plotted in Figure 2. The *P* value from a stratified log-rank test was also not significant (*P*=0.93).

There was a tendency for a good neurological outcome (CPC 1 and 2) at discharge to be present more often in the thiamine group than in the placebo group, but the difference was not statistically significant (35% vs. 23.5%; RR 1.27, 95% CI 0.71–2.23, *P*=0.45).

The laboratory parameter outcome was similar between the two groups. The thiamine group tended to have lower S100B levels and higher lactate clearance in 24 and 48 h compared with the placebo, but the difference was not statistically significant.

In all, 51.4% of patients (19/37) survived to day 28. Surviving patients had a lower CPR time, APACHE II score, and S100B at 72 h (Table 3).

### 3.3. Adverse Events

There were no reports of major adverse events related to thiamine administration. Only one patient in the thiamine group developed a maculopapular rash during admission, which was diagnosed as a phenytoin allergy. In-hospital complications between the two groups are shown in Table 1, with no significant differences.

## 4. Discussion

This study evaluated the effect of thiamine in comatose adult OHCA patients. The results of the trial did not show a significant difference between the two groups in the primary endpoints of all-cause 28-day mortality and neurological outcomes at hospital discharge.

Our findings did not corroborate the findings of a previous animal study [9], which showed a survival benefit of thiamine administration in mice with cardiac arrest. The difference in results could be related to the protocol of the animal study, whereby cardiac arrest was induced in mice during anesthesia and immediate resuscitation started by the investigators. In contrast, in our study, OHCA patients were administered CPR by bystanders, with only 51% leading to prolonged resuscitation. The numbers of witnesses to cardiac arrest and bystander CPR in this study were not different from those in our previous report. Bystander CPR was strongly associated with survival in OHCA [13].

Although our institution adopts the latest guidelines for postcardiac arrest care, our OHCA patients received coronary angiography only 18/37 (48.6%) times and targeted temperature management only 16/37 (43.2%) times. These two interventions were related to survival and neurological outcome. These findings are correlated with our previous study [14] and reflect real practices in most Asian countries [15].

Another explanation for the difference between the results of our trial and previous animal studies is the dosage and timing of thiamine administration. In an animal study [9], thiamine hydrochloride 50 mg/kg was administered 2 min before CPR and followed by daily intraperitoneal injection. In our study, thiamine was administered in doses of 300 mg/day after the patients had successful ROSC based on the prophylaxis dosage regimen for patients with suspected Wernicke encephalopathy [16]. The median time from cardiac arrest to drug administration was 3.5 h.

A previous study showed that thiamine could improve PDH activity after cardiac arrest [9]. In our study, we measured thiamine levels after patients had ROSC, instead of PDH activity, and found a median level of 93 (IQR, 80.0–122.0) nmol/L. Using the cut-off value of plasma thiamine levels ≤9 nmol/L [17], no patient was diagnosed with absolute thiamine deficiency. No information is available on thiamine levels in postcardiac arrest patients. We suspect that the level of serum thiamine in postcardiac arrest patients might be varied and unpredictable due to the unstable physiology of patients. In our multivariate analysis model, thiamine levels were not associated with in-hospital mortality.

Thiamine is used as adjunct to standard treatment in sepsis resuscitation and cardiac surgery. Thiamine significantly decreased lactate levels, but only in thiamine-deficient septic shock patients [18]. In cardiac surgery, thiamine administration did not show any benefit in lactate clearance [19].

We used S100B and lactate levels as secondary endpoints. The S100B level was used to predict neurological prognosis in postcardiac arrest patients [20, 21]. Besides S100B, the lactate level is also a neurological marker [22]. Thiamine administration did not alter S100B and lactate levels in our study. However, we found that the thiamine group trended toward having better lactate clearance at 24 and 48 h. The lactate clearance outcomes should be emphasized and explored in further studies.

Our study reported a 28-day mortality rate of 48.6% in all participants, similar to another study in Thailand [10]. The APACHE II score at ICU admission was significantly lower in the survival group. This parameter should be stratified before enrollment, because thiamine administration might be of benefit depending on the severity of patients.

The results of our study should be interpreted in the context of several limitations. First, this study is small and in a single center that lacks the power to show statistical significance. Second, only half of our OHCA patients were given CPR by bystanders, and CPR time was longer than usual, which may have affected neurological outcomes and survival. Third, the dosage and duration of thiamine administration were determined by the investigator, and we did not determine the benefits of a different dosage, timing, and duration.

Future studies are needed to confirm the benefits of thiamine in terms of neurological outcomes and survival.

## 5. Conclusions

In patients who had been successfully resuscitated after OHCA and were comatose, administration of thiamine hydrochloride 100 mg intravenously every 8 h for 7 days did not show a survival benefit or improve neurological outcomes.

## Figures and Tables

**Table 1:** Baseline characteristics of out-of-hospital cardiac arrest (OHCA) patients randomized to receive thiamine or placebo.

Characteristics	Thiamine (*n* = 20)	Placebo (*n* = 17)
Age, mean (SD) (y)	69.1 (13.9)	63.1 (14.2)
Male gender	14 (70)	12 (70.6)
Comorbidities		
Hypertension	10 (50)	9 (52.9)
Diabetes mellitus	3 (15)	4 (23.5)
Coronary artery disease	5 (25)	2 (11.8)
Others	10 (50)	13 (35.1)
Witnessed arrest	19 (95)	17 (100)
Bystander CPR	9 (45)	10 (58.8)
Shockable rhythm	11 (55)	8 (47.1)
Nonshockable rhythm	9 (45)	9 (52.9)
Time from cardiac arrest to event, median (IQR) (min)		
Start of BLS	5 (1–22)	5 (1–15)
Start of ACLS	12 (2–30)	10 (5.5–18.5)
ROSC	28.5 (8–46.5)	23 (14.5–34)
CPR time, median (IQR) (min)	13 (6.5–30)	14 (9–23.5)
Number of defibrillation attempts, median (IQR)	1 (0–3)	2 (0–3)
Adrenaline doses, median (IQR) (mg)	3 (2–6)	3 (2–6)
Causes of cardiac arrest		
Cardiac causes	14 (70)	12 (70.6)
STEMI^a^	11 (78)	3 (25)
Other causes	6 (30)	5 (29.4)
Time from admission to receiving intervention drugs, median (IQR) (h)	3.5 (2–5.7)	2 (1–4)
GCS	6 (3–7)	5 (3–8)
Circulatory shock^b^	20 (100)	14 (82.4)
APACHE II, mean (SD)	25.1 (6.4)	25.8 (8.1)
Thiamine level (nmol/L)	91 (82.2–125.8)	98 (70–113.5)
Lactate level (mmol/L)	7.2 (3.5–10.8)	7.1 (4.8–10.7)
S100B level (*μ*g/L)	0.33 (0.159–0.719)	0.659 (0.173–2.155)
Procedures		
Coronary angiography	11 (55)	7 (41.2)
Percutaneous coronary intervention	7 (35)	4 (23.5)
Targeted temperature management	8 (40)	8 (47.1)
Complications		
VAP	9 (45)	7 (41.2)
AKI	18 (90)	16 (94.1)

Data are presented as numbers (%) unless otherwise specified. ^a^Significant with *P*=0.02. ^b^Circulatory shock was defined as a systolic blood pressure of <90 mmHg for >30 min or end organ hypoperfusion (i.e., cool extremities, urine output < 30 mL/h, and heart rate < 60 beats/min). ACLS, advanced cardiovascular life support; AKI, acute kidney injury; APACHE II, Acute Physiology and Chronic Health Evaluation II; BLS, basic life support; CPR, cardiopulmonary resuscitation; GCS, Glasgow Coma Scale; IQR, interquartile range; ROSC, return of spontaneous circulation; SD, standard deviation; STEMI, ST-segment elevation myocardial infarction; VAP, ventilator-associated pneumonia

**Table 2:** Primary and secondary outcomes of out-of-hospital cardiac arrest (OHCA) patients receiving thiamine or placebo.

Outcome	Thiamine (*n* = 20)	Placebo (*n* = 17)	Relative risk (95% CI)	*P* value
Primary outcome				
28-day mortality	10 (50)	8 (47.1)	1.06 (0.54–2.07)	0.86
Secondary outcome				
Good neurological outcome (CPC 1 or 2 at discharge)	7 (35)	4 (23.5)	1.27 (0.71–2.23)	0.45
S100B level at 72 h^a^ median (IQR) (ng/L)	0.126 (0.085–0.349)	0.168 (0.073–0.987)	—	0.81
Lactate level at 24 h, median (IQR) (mmol/L)	2.3 (1.5–5.6)	2.6 (2–4.4)	—	0.35
Lactate level at 48 h, median (IQR) (mmol/L)	2 (1.5–3.7)	2 (1.4–2.2)	—	0.76
Lactate level at 72 h^b^, median (IQR) (mmol/L)	1.6 (1.1–2.5)	1.5 (1–1.8)	—	0.24
Lactate clearance at 24 h, median (IQR) (%)	59.7 (34.1–80.2)	53.3 (41.5–69.6)	—	0.52
Lactate clearance at 48 h, median (IQR) (%)	75.2 (34.1–80.8)	67.1 (57.2–74.4)	—	0.70
Lactate clearance at 72 h, median (IQR) (%)	74.8 (47.5–84.4)	75 (64.4–82.8)	—	0.69
ICU length of stay (days)	7 (5–9)	5 (3–7)	—	0.18

^a^S100B levels at 72 h were collected in 19 patients from the thiamine group and 12 patients from the placebo group. ^b^Lactate levels at 72 h were collected in 16 patients from the thiamine group and 12 patients from the placebo group. Some patients either did not have a second or third lactate sampling or the results were missing or otherwise unavailable. Data are presented as medians (IQR) unless otherwise specified. CI, confidence interval; CPC, cerebral performance category; ICU, intensive care unit; IQR, interquartile range.

**Table 3:** Characteristics of out-of-hospital cardiac arrest (OHCA) patients receiving thiamine or placebo categorized into death and survival at 28 days.

Characteristic	Survived (*n* = 19)	Death (*n* = 18)	*P* value
Age (y)	68.0 (53.0–74.0)	68.5 (58.8–80.0)	0.41
CPR time (min)	8 (6–23)	16 (10.0–31.25)	0.03
Time from arrest to ROSC (min)	18 (8–30)	32.5 (14.8–48.0)	0.28
Adrenaline doses (mg)	3 (2–5)	3 (2.8–8)	0.49
STEMI, *n* (%)	7 (36.8)	7 (38.9)	0.16
APACHE II	23.0 (17.0–28.0)	26.5 (24.8–32.0)	0.01
Thiamine level (nmol/L)	98 (64–125)	92 (82.0–120.8)	0.72
Initial lactate level (mmol/L)	5.9 (4.8–10.5)	8.0 (4.1–11.0)	0.98
Lactate level at 24 h (mmol/L)	2.4 (1.7–5.3)	2.4 (1.8–5.5)	0.86
Lactate level at 48 h (mmol/L)	2.0 (1.4–2.6)	1.9 (1.6–3.5)	0.97
Lactate level at 72 h, mmol/L	1.5 (1.0–2.2)	1.6 (1.2–2.1)	0.35
Initial S100B level (*μ*g/L)	0.28 (0.14–0.80)	0.52 (0.22–3.18)	0.24
S100B level at 72 h (*μ*g/L)	0.09 (0.06–0.18)	0.28 (0.18–0.45)	<0.01

Data are presented as medians (IQR) unless otherwise specified. APACHE II, Acute Physiology and Chronic Health Evaluation II; CPR, cardiopulmonary resuscitation; IQR, interquartile range; ROSC, return of spontaneous circulation; STEMI, ST-segment elevation myocardial infarction.

## Data Availability

The data used to support the findings of this study are available from the corresponding author upon request.

## Supplementary Materials

Supplementary Materials Table S1: baseline characteristics of out-of-hospital cardiac arrest (OHCA) patients randomized to receive thiamine or placebo. Table S2: procedures, interventions, and medications during ICU admission.
