# Supplementary material for: Efficacy of Thiamine in the Treatment of Postcardiac Arrest Patients: A Randomized Controlled Study
Source: Crit Care Res Pract. 2020 Jun 8;2020:2981079. doi: 10.1155/2020/2981079 (PMC7298263; doi:10.1155/2020/2981079)
Supplement: Supplementary Materials — Table S1: baseline characteristics of out-of-hospital cardiac arrest (OHCA) patients randomized to receive thiamine or placebo. Table S2: procedures, interventions, and medications during ICU admission. [file 2981079.f1.pdf]

Supplementary Appendix

Independent data monitoring and committee

The independent data monitoring and committee is under the Office of Human Research Ethics Committee (HREC), Faculty of Medicine, Prince of Songkla University. The primary role is to protect patient safety. The investigators must report all adverse events that are possibly related to the study and all patients that died during the study.

Discontinued criteria

1. Patients or representative withdrawing.
2. Presence of side effects of thiamine administration.

Inclusion and exclusion criteria

Inclusion criteria

1. Patients aged 18 years or older.
2. Successfully resuscitated from out-of-hospital cardiac arrest (OHCA) and sustained return of spontaneous circulation (ROSC) (more than 20 consecutive min and signs of circulation persist).
3. Patients who were comatose (Glasgow coma score of 8 or less).
4. Patients were admitted to the medical intensive care unit (ICU).

Exclusion criteria

1. Known limitation in therapy or Do Not Resuscitate order.
2. Pregnancy.
3. Suspected or confirmed intracranial pathology (ischemic stroke or intracerebral hemorrhage).
4. Poor neurological performance status (pre-arrest cerebral performance category [CPC] 3 or 4).
5. Prior cardiac arrest.
6. Contraindications to thiamine.

Supplementary tables

Table S1 Baseline characteristics of out-of-hospital cardiac arrest (OHCA) patients randomized to receive thiamine or placebo.

**Characteristic**	**Thiamine (n = 20)**	**Placebo (n = 17)**
The first person contacted by a bystander		
Relative	6 (30)	7 (41.2)
EMS	11 (55)	7 (41.2)
Hospital	2 (10)	1 (5.8)
No call	1 (5)	2 (11.8)
First monitored cardiac rhythm		
Asystole	3 (15)	2 (11.8)
PEA	6 (30)	7 (41.2)
VF	10 (50)	8 (47.1)
VT	1 (5)	0
Defibrillated by EMS	4 (20)	5 (29.4)
AED use by bystander or EMS	5 (25)	5 (29.4)
EMS response time, median (IQR), min	20 (8–30)	10 (5–22.5)
Mode of transportation		
Own transport	8 (40)	7 (41.2)
EMS	8 (40)	10 (58.8)
Public transport	4 (20)	0
Cause of cardiac arrest		
Cardiac causes	14 (70)	12 (70.6)

Characteristic	Thiamine<br><br>(n = 20)	Placebo<br><br>(n = 17)
STEMI	11 (78)	3 (25)
Respiratory failure	5 (25)	4 (23.5)
Metabolic causes	1 (5)	0
Unidentified causes	0	1 (5.9)
Hospital stay, median (IQR), days	15 (8–33)	11 (3.5–29.5)

Notes: Data are presented as numbers (%) unless otherwise specified.
Abbreviations: AED, automated external defibrillator; EMS, emergency medical service; IQR, interquartile range; PEA, pulseless electrical activity; STEMI, ST-segment elevation myocardial infarction; VF, ventricular fibrillation; VT, ventricular tachycardia

Table S2 Procedures, interventions, and medications during ICU admission.

Variable	Thiamine (n = 20)	Placebo (n = 17)
CAG		
Number of patients	11 (55)	7 (41.2)
Found culprit lesions	7 (35)	4 (23.5)
LAD	5 (71.4)	1 (25)
RCA	2 (28.6)	3 (75)
Successful PCI	7 (35)	3 (17.6)
TTM		
Number of patients	8 (40)	8 (47.1)
Time from ROSC to start TTM, median (IQR), h	3 (2–6.5)	4 (4–6)
Induction time, median (IQR), h	4 (4–5)	4 (3.8–6)
Maintenance time, median (IQR), h	24 (24–24)	24 (24–24)
Rewarming time, median (IQR), h	12 (9–13)	12 (9–14)
IABP	2 (10)	2 (11.8)
Dopamine	5 (25)	6 (35.3)
Epinephrine	4 (20)	5 (29.4)
Norepinephrine	7 (35)	6 (35.3)

Notes: Data are presented as numbers (%) unless otherwise specified.
Abbreviations: CAG, coronary angiography; IABP, intra-aortic balloon pump; IQR, interquartile range; LAD, left anterior descending artery; PCI, percutaneous coronary intervention; RCA, right coronary artery; TTM, targeted temperature management
